# Atraumatic Left Distal Radial Artery Aneurysm

**DOI:** 10.1155/2019/4608171

**Published:** 2019-09-05

**Authors:** Joseph Maalouly, Dany Aouad, Elias Saidy, Antonios Tawk, Georges Baaklini, Chawki Cortbawi

**Affiliations:** ^1^Department of Orthopedic Surgery and Traumatology, Saint Georges University Medical Center, Balamand University, P.O.Box 166378, Achrafieh, Beirut 1100 2807, Lebanon; ^2^Saint Georges University Medical Center, Balamand University, P.O.Box 166378, Achrafieh, Beirut 1100 2807, Lebanon; ^3^Department of Vascular Surgery, Saint Georges University Medical Center, Balamand University, P.O.Box 166378, Achrafieh, Beirut 1100 2807, Lebanon

## Abstract

Distal radial artery aneurysms are an uncommon pathological entity in the field of surgery. Moreover, distal radial artery aneurysms of idiopathic etiology are even rarer. Herein, we present a rare case of idiopathic/atraumatic left radial artery aneurysm. A 73-year-old female patient presented with a chief complaint of a pulsatile mass located on her left wrist. Radiological imaging showed the presence of a distal radial artery aneurysm which was successfully surgically excised with subsequent ligation of the radial artery. Some of the etiologies and operative management of distal radial artery aneurysm in the anatomical snuffbox to what is in accordance with the literature are discussed. Distal radial artery aneurysms are rare. Hence, their misdiagnosis may lead to wrongful management and increase in morbidity. The appropriate management of distal radial aneurysm is almost always surgical.

## 1. Introduction

Arterial aneurysms of the upper extremity form an extremely rare pathological entity encountered in the surgical field. The rarest form of upper extremity arterial aneurysm is the distal radial artery aneurysm, while the most common form is the distal ulnar artery aneurysm. The majority of radial artery aneurysm cases reported in the literature are secondary to a traumatic event, with the anatomical snuffbox being the most common anatomical location [1, 2]. Other upper extremity arterial aneurysm etiologies reported in the literature include mycotic [3], arteriosclerotic [4, 5], idiopathic [6], and underlying vasculopathy [7]. The rarest etiology of radial artery aneurysm is the idiopathic etiology accounting for only nine cases in the literature [8]. A true aneurysm is when there is dilatation of the artery, usually occurring secondary to weakening of the arterial wall. Thus, the dilatation contains all the components of the arterial wall. A false aneurysm or a pseudoaneurysm is when there is a protrusion of a sac-like structure from an otherwise normal artery. Pseudoaneurysms are usually secondary to a disruption of the arterial wall [9]. Herein, we report a case of idiopathic distal radial artery aneurysm.

## 2. Case Presentation

**Figure 1:**
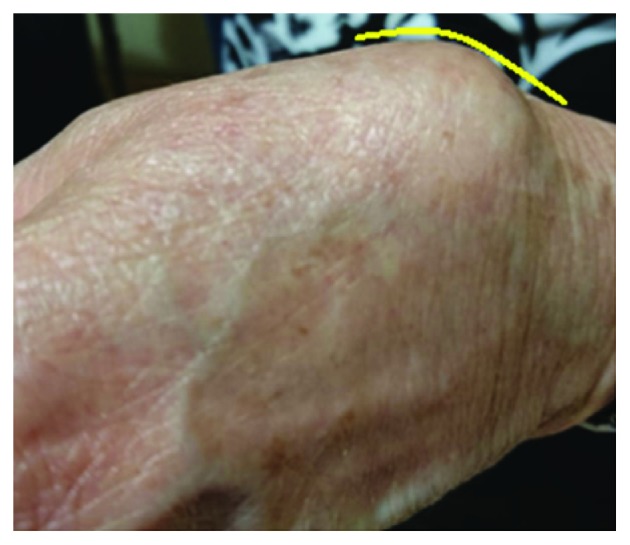
A mass located at the snuffbox area of the left hand.

A 73-year-old female patient with a past medical and surgical history positive for hypertension, dyslipidemia, and thrombocytopenia status postsplenectomy presented with a chief complaint of a pulsatile mass located on her left wrist. The patient denied any family history of aneurysms, any traumatic event to the hand, any recurrent punctures or arterial cannulation, and any surgeries. The patient reported that she is a nonsmoker. The patient started noticing the mass one year prior to her presentation. She describes her mass as small at the beginning and gradually increasing in size. Upon physical examination, we noticed an irregular dark grey focally congested membranous fragment associated with a separate rubbery to firm beige tissue fragment (Figure 1). The mass was pulsatile, but no bruit was heard upon auscultation. The finding on Allen's test was positive. Upon further full body inspection and auscultation, no evidence of other aneurysms in other parts of the body was found. A laboratory workup was preformed, and results were negative for any evidence of systemic inflammation or disease of autoimmune etiology.

**Figure 2:**
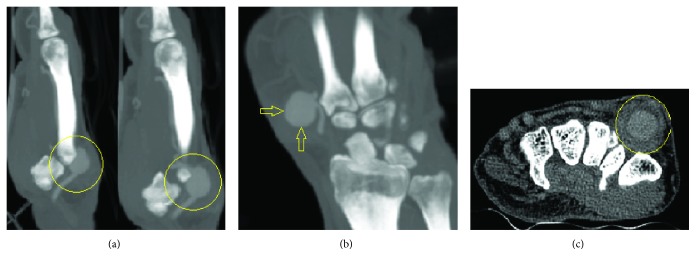
Computed Tomography Angiography shows the 15 × 9 × 11 mm distal radial artery aneurysm (yellow circles and arrows) in sagittal (a), coronal (b), and transverse (c) sections.

Computed Tomography Angiography (CTA) with IV contrast showed a 15 × 9 × 11 mm aneurysm of the distal radial artery as it forms the dorsal arch (Figure 2). The radial and ulnar arteries were normally opacified and patent. There was no evidence of blood supply compromise at the level of the hand with intact superficial and deep palmar arches. The CT scan showed mild degenerative changes of the radioulnar joint, radiocarpal joint, and first carpometacarpal joint.

However, there were no evidence of trauma, previous fractures, or bone lesions.

Taking into consideration of various complications of an aneurysm from a thromboembolic event to rupture, surgical intervention was decided. The surgery was a joint surgery between the vascular surgery department and the orthopedic surgery department.

**Figure 3:**
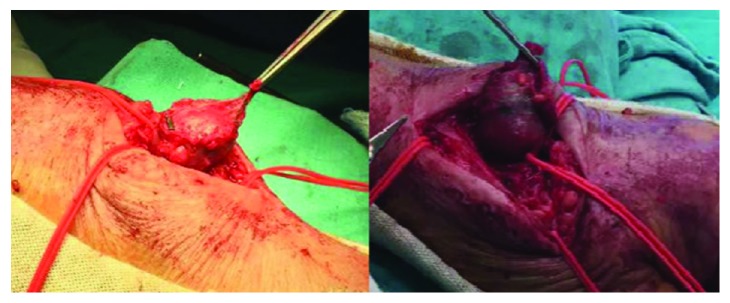
Exploration of the aneurysm with the radial artery controlled with vessel loops proximally and distally.

Under local anesthesia, the surgeons made an incision at the aneurysm site and established a primary control of the proximal and distal artery to the aneurysm using vessel loops. The aneurysm was dissected from the surrounding neurologic structures, and the artery was clamped (Figure 3). The aneurysm was then excised, and primary repair with end-to-end anastomosis was done.

The patient was followed postoperatively for two days before her discharge with an uneventful hospital stay.

The histopathology report of the excised mass described features compatible with an aneurysm filled with nonorganized fibrin thrombus. The report also described the aneurysm macroscopically as an irregular, dark-grey, focally congested membranous tissue fragments measuring 3 × 1 × 0.7 cm associated with a separate rubbery to firm beige tissue fragment measuring 1.3 × 1 × 0.2 cm.

## 3. Discussion

Upper extremity aneurysms are extremely rare encounters in the surgical field with radial aneurysms being the rarest with a prevalence of 2.9% among all aneurysms affecting the upper extremities [2]. This rarity is attributed to the fact that the radial artery has a small lumen. Thus, there is a low probability for an aneurysm to form based on Laplace's law since vessels with small lumen require higher pressures for the aneurysm to enlarge [9, 10]. The most common location for a distal radial artery aneurysm is at the level of the anatomical snuffbox.

In this presented case, our patient presented with a chief complaint of a pulsatile mass located on her left forearm with no associated pain or paresthesia. The diagnosis of the distal radial artery aneurysm was made clinically with physical exam findings of a pulsatile mass along with radiologic modalities used for the visualization of the mass. The diagnosis was later confirmed with postoperative pathological studies. Due to the potential complications of an aneurysm, surgical intervention was advised as the appropriate management of the distal radial artery aneurysm. Rupture of the aneurysm is a rare complication. Other complications include thromboembolism formation with subsequent distal ischemia due to vessel occlusion or nerve compression due to expanding mass [6, 11]. Based on physical exam and imaging finding, a reconstructive approach was taken. The aneurysm was surgically excised with subsequent radial artery ligation via a primary end-to-end anastomosis. The postoperative course of the patient was uneventful, and the patient was discharged two days postoperation.

## 4. Conclusion

Atraumatic or idiopathic distal radial artery aneurysms are extremely rare encountered medical conditions. The misdiagnosis of an upper extremity aneurysm for a ganglion, a neuroma, a lipoma, or a synovial cyst leads to wrongful management, and it is associated with increased morbidity. The appropriate diagnosis of a distal radial artery aneurysm in the above patient was the combination of the clinical picture painted with the patient's history and physical examination and the radiologic modalities used for better visualization. Due to the various complications of an upper extremity aneurysm that can range from a thromboembolic event to rupture, surgery is usually indicated as it is the case with our patient. The operative management of our patient included the surgical excision of the distal radial artery aneurysm on the left upper extremity with subsequent ligation of the radial artery via end-to-end anastomosis.

However, which surgical approach to use remains controversial as the literature does not contain clear guidelines for the operative management of such pathology since distal radial artery aneurysm at the anatomical snuffbox are uncommon entities reported as case reports in the literature [8, 12, 13].
